# James Guy Edwards (Guy), FRCPsych, FRCP, DPM, MFPH (Hon.)

**DOI:** 10.1192/bjb.2023.5

**Published:** 2023-08

**Authors:** Ben Steinberg, Philip Graham

Formerly Consultant Psychiatrist, Southampton University Hospitals, Southampton, UK

**Figure:**
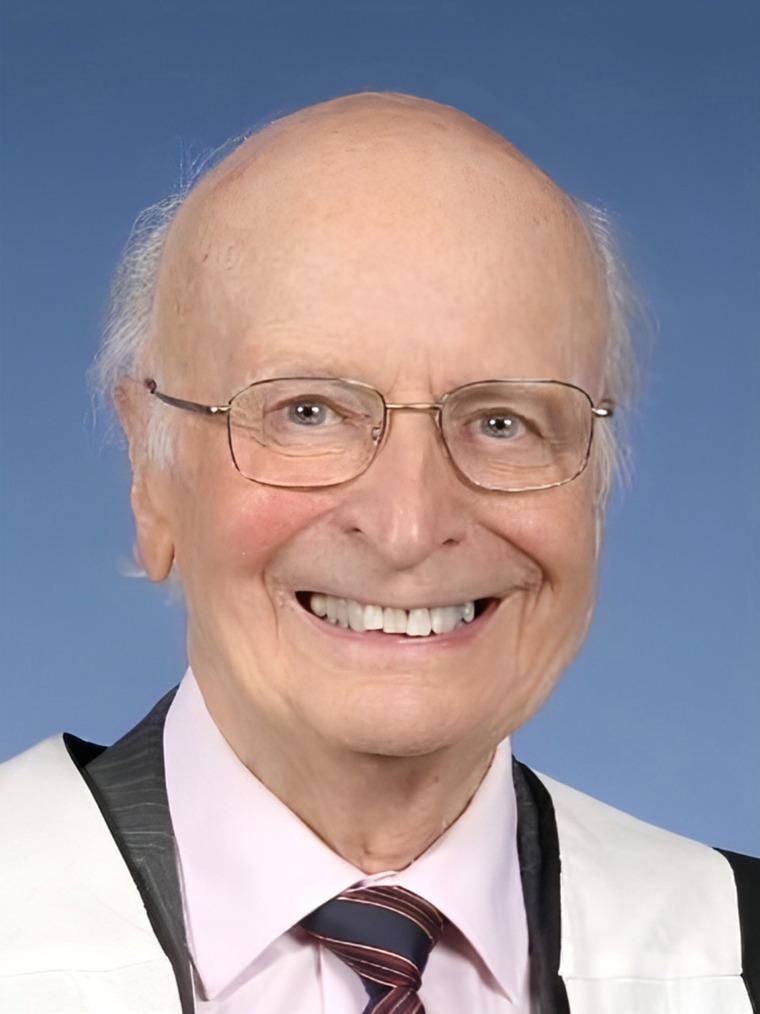

Guy Edwards, who died on 4 November 2022 at the age of 88, combined full-time clinical practice with a productive research career. He published over 180 papers, mainly on clinical aspects of psychotropic medication, especially antidepressants. He would not accept that there was ‘no time for research’; he made time for it – by spending an hour per day on a research-related topic, collaborating with colleagues in psychiatric and non-psychiatric specialties and obtaining small grants for studies that could be incorporated into his full-time clinical work without interfering with it. As he was first and foremost a clinician, Guy used to say that he felt like ‘a trespasser into academia’, but one distinguished academic referred to him as ‘one of the most academic non-academics in the country’ – a well-deserved compliment.

He also carried out a significant number of editorial responsibilities. He was the founder and Editor-in-Chief of the journal *Human Psychopharmacology*, a post he filled with distinction from 1985 to 1993. He also served as Honorary Treasurer of the British Association for Psychopharmacology (1984–1989). He was involved in a considerable number of teaching and research activities abroad at various universities, including in Nigeria, Japan and Thailand. His links with Thailand were particularly close in the later stages of his career.

Guy was born in a mining village in South Wales, the only child of James Edwards, part-owner of a coach business, and Dilys May Edwards (née Williams), who ran a shop. His father died when he was 7 years old. Guy was the only one of his extended family who at the time had a secondary school education. He graduated from the Welsh National School of Medicine in Cardiff and, after completing his house jobs in surgery, medicine and neurology, he obtained a year's experience as a general practitioner and assistant ship's surgeon, and then embarked on a psychiatric career. He began his psychiatric training in Manchester, where he was influenced by Professor E.W. Anderson's phenomenological approach to psychiatry, and then worked as a registrar in Guy's Hospital, London.

After completing his psychiatric training in 1964, Guy worked for 3 years as a research psychiatrist in the Rockland Research Institute and College of Physicians and Surgeons of Columbia University, New York, and a staff psychiatrist in Bergen Pines County Hospital, New Jersey, USA, before returning to the UK. He was then appointed consultant psychiatrist at Knowle Hospital, Fareham, which was affiliated to the Southampton University Medical School. He played a very active part in the development of local psychiatric services, first in the closure of Knowle Hospital and then in the Department of Psychiatry, Royal South Hants Hospital, Southampton. Here, with his colleagues he developed a community and domiciliary service for Southampton and the South West of England, eventually resigning from the National Health Service in 1993.

Nationally, he was an Examiner for the MRCPsych examination for 10 years and later an Observer (quality assurance assessor of the standard of examiners and examination centres) for 20 years. He also examined for the United Examining Board of the Royal College of Physicians and Surgeons and Society of Apothecaries (1993) and occasionally for the University of London as an external examiner of PhD students (1989–1993). Early in his consultant career Guy was seconded to the Department of Health's Health Advisory Service. Later, he served as Medical Member on Mental Health Review Tribunals (sitting at Broadmoor and hospitals in the south of England) for almost 20 years and as Physician on Medical Appeals Tribunals. He was a Health Examiner for the General Medical Council for 33 years, latterly being appointed Specialist Health Advisor to the General Medical Council/General Practitioners’ Tribunal Service.

In recognition of his lifelong contributions to psychopharmacology and psychiatry he was elected a Distinguished Fellow of the Royal College of Physicians of London.

In both his professional and private life Guy was a reserved person but with a great sense of humour. Despite his achievements he was modest in manner, with a complete absence of pomposity. Because of his basic shyness many people did not really get to know him and in a few cases may indeed have misunderstood him. To those of us who were his friends – and he had many – he came across as a warm, kind, generous person. He was witty and had a large fund of jokes, Welsh and others. Many people experienced his generosity and genuine friendliness. He had indeed a very colourful personality and will be greatly missed.

He married Althea (née Pym) in 1973. They divorced 35 years later. Guy was deeply devoted to his family and left three children – Dafydd, Judy and Nicola – and three grandchildren.

